# An optimized protocol for generating appendage-bearing skin organoids from human-induced pluripotent stem cells

**DOI:** 10.1093/biomethods/bpae019

**Published:** 2024-03-22

**Authors:** Imaan Ahmed, Jane Sun, Jason Brown, Kiarash Khosrotehrani, Abbas Shafiee

**Affiliations:** The University of Queensland Frazer Institute, The University of Queensland, Brisbane, QLD 4102, Australia; The University of Queensland Frazer Institute, The University of Queensland, Brisbane, QLD 4102, Australia; Herston Biofabrication Institute, Metro North Hospital and Health Service, Brisbane, QLD 4029, Australia; Royal Brisbane and Women’s Hospital, Metro North Hospital and Health Service, Brisbane, QLD 4029, Australia; The University of Queensland Frazer Institute, The University of Queensland, Brisbane, QLD 4102, Australia; Herston Biofabrication Institute, Metro North Hospital and Health Service, Brisbane, QLD 4029, Australia; The University of Queensland Frazer Institute, The University of Queensland, Brisbane, QLD 4102, Australia; Herston Biofabrication Institute, Metro North Hospital and Health Service, Brisbane, QLD 4029, Australia; Royal Brisbane and Women’s Hospital, Metro North Hospital and Health Service, Brisbane, QLD 4029, Australia

**Keywords:** disease modelling, sweat gland, regenerative medicine, stem cell, hair follicle, organoid

## Abstract

Organoid generation from pluripotent stem cells is a cutting-edge technique that has created new possibilities for modelling human organs *in vitro*, as well as opening avenues for regenerative medicine. Here, we present a protocol for generating skin organoids (SKOs) from human-induced pluripotent stem cells (hiPSCs) via direct embryoid body formation. This method provides a consistent start point for hiPSC differentiation, resulting in SKOs with complex skin architecture and appendages (e.g. hair follicles, sebaceous glands, etc.) across hiPSC lines from two different somatic sources.

## Introduction

Skin is a highly complex organ, with important physiological roles in barrier protection, thermoregulation, and sensation [1]. With so many crucial roles, it follows that skin pathologies, such as inflammatory conditions and deep burns, can have devastating impacts on wellbeing. Generating human skin models *in vitro*, therefore, is a research area of great importance. An effective skin model would have multiple applications, including disease modelling, developmental studies, drug testing, and regenerative medicine purposes. Furthermore, human skin models are necessary to reduce the need for and overcome the limitations associated with animal skin models.

Existing methods of generating human skin *in vitro* have, until very recently, been limited in their ability to recapitulate the functional organ [2]. Bilayer skin equivalent models, for example, succeed in recapitulating the stratified squamous epithelium of the epidermis and the underlying fibroblasts of the dermis, but due to the simplistic co-culture approach, fail to generate any skin appendages (i.e. hair follicles, sweat glands, and sebaceous glands) [3, 4].

In 2020, however, Lee *et al*. developed a new method to generate skin organoids from human-induced pluripotent stem cells (hiPSCs) [5]. hiPSCs are embryonic-like stem cells with the potential to differentiate into any cell type of the human body. By aggregating hiPSCs, maintaining the aggregates for 48 h, then performing stepwise modulation of the TGFβ/BMP pathway which drives embryonic skin development, Lee *et al*. successfully generated human skin organoids (SKOs). Unlike previous skin models, mature SKOs possess not only an epidermis and dermis, but also neurites, Merkle cells, sebaceous glands, and hair follicles. However, this protocol produced variability between hiPSC lines [5, 6].

We aimed to optimize the SKO differentiation protocols by minimizing the hiPSC line dependency effect [7]. The original SKO protocol aggregates 3500 hiPSCs in maintenance media and cultures for 48 h, which may result in differently sized aggregates due to different hiPSC line proliferation rates, and subsequently require hiPSC line-dependent optimization of the differentiation reagents. Here, we instead aggregate 8000 hiPSCs in E6 differentiation media, to initiate embryoid body formation prior to performing SKO differentiation 24 h later. We hypothesized that this shortened differentiation process could create more consistently sized aggregates across hiPSC lines, and thereby improve the consistency of SKO differentiation. Indeed, we have found our protocol to be successful in all three hiPSC lines we have tested, whereas the original protocol failed in the C32 hiPSC line in our hands [7], and in the WA09 line used in the original paper [5]. Additionally, we present an improved method to transfer SKOs between culture medias, for technical ease.

## Materials

### Antibodies

- Mouse polyclonal anti-beta III tubulin antibody (TUJ1), BioLegend, 801201
- Mouse monoclonal anti-cytokeratin-10 antibody, Abcam, ab9026
- Mouse monoclonal anti-P-cadherin antibody, ThermoFisher, 32-4000
- Rabbit monoclonal anti-cytokeratin-14 antibody, Abcam, ab181595
- Rabbit monoclonal anti-cytokeratin-17 antibody, Abcam, ab109725
- Donkey anti-rabbit IgG (H+L) Highly Cross-Adsorbed Secondary Antibody, Alexa Fluor 568, ThermoFisher, A10042
- Goat anti-mouse IgG (H+L) Highly Cross-Adsorbed Secondary Antibody, Alexa Fluor 488, ThermoFisher, A11029

### Reagents

- ROCK inhibitor Y27632, Miltenyi Biotec, 130-106-538
- Antibiotic-Antimycotic, ThermoFisher, 15240096
- mTeSR Plus, Stemcell Technologies, 5825
- DMEM/F12, ThermoFisher, 1132003
- StemPro Accutase Cell Dissociation Reagent, ThermoFisher, A1110501
- Matrigel hESC-Qualified Matrix, Corning, 354277
- Matrigel Growth Factor Reduced Basement Membrane Matrix, Corning, 354230
- SB43152 (SB), Stemgent, 04-0010-05
- Recombinant Human BMP-4 (BMP), Lonza Bioscience, 120-05ET-50
- Recombinant Human FGF-basic (FGF2), Lonza Bioscience, 100-18B
- LDN193189 (LDN), Lonza Bioscience, 1066208-A
- Essential 6, ThermoFisher, A1516401
- Advanced DMEM/F12, ThermoFisher, 12634010
- Neurobasal Medium, ThermoFisher, 21103049
- GlutaMAX Supplement, ThermoFisher, 35050061
- B-27 Supplement Minus Vitamin A, ThermoFisher, 12587010
- N-2 Supplement, ThermoFisher, 17502048
- 2-Mercaptoethanol, ThermoFisher, 21985023
- DAPI, ThermoFisher, D1306
- Normal Goat Serum, ThermoFisher, 31873
- Dulbecco’s phosphate-buffered saline (DPBS), ThermoFisher, 14190-144

### Cell lines

- C32 iPSCs
- P111 iPSCs

### Software

- FIJI/Image J, Version 2.1.0/1.53c
- NIS-Elements Advanced Research software, Version 5.11

### Equipment

- Bright-Line Hemacytometer, Sigma-Aldrich, Z359629
- Class II Biological Safety Cabinet (Biosafety Cabinet), ESCO, LA2-4K1
- Incubator with Segmented Inner Doors, Binder, CB220
- Tissue Culture Incubator, Panasonic, MCO-170AIC
- Inverted Microscope for tissue culture, Olympus, CKX41
- Brightfield Microscope for imaging, Olympus, IX73
- Ti-E Spectral Spinning Disk Confocal, Nikon/Yokogawa, CSU-21
- Ultra-Low Attachment 96-Well Plate, Corning, 7007
- Ultra-Low Attachment 24-Well Plate, Corning, 3473
- 6-Well Plate, Corning, 3516
- Axygen Axypet Pipettors (1000, 200, and 10 μl), Corning, AP-1000, AP-200, AP-10
- Vertex Pipette Tips (1000, 200, and 10 μl), SSI Bio, 4337NSF, 4237NSF, 4137NSF
- Multichannel Reagent Reservoirs, Integra, 4331
- 0.2 μm Rapid-Flow Filter Unit, ThermoFisher, 566-0020

### Media recipes

*Note*: It is recommended to obtain the growth factors used in the differentiation media from the same companies that have been used in this study, as we have demonstrated that these are effective for this protocol.

## Methods

### hiPSC maintenance

*Note:* This section outlines hiPSC maintenance in single cell format, using mTeSR Plus medium with a Human Embryonic Stem Cell-Qualified Matrigel (hESC Matrigel) coating. However, this is not the exclusive way to culture hiPSCs for SKO differentiation. Alternative hiPSC culture methods can be used, such as Essential 8 medium with a vitronectin coating.

*Note:* All steps relating to cell culture should be performed in a Class II biosafety cabinet.

#### Timing: ∼1 h

1. Coat a 6-well cell culture plate with hESC-Qualified Matrigel
  - For each well to be coated, dilute hESC-Qualified Matrigel to 1× in 1 ml cold DMEM/F12 in a 15 ml Falcon tube
  - Pipette 1 ml of diluted Matrigel in DMEM/F12 per well immediately into six-well plate to be coated, swirling the plate to ensure the bottom of the wells are fully covered
  - Incubate for at least 30 min at 37°C (can be incubated for longer but no more than 8 h).
2. Thaw out a cryovial of hiPSCs
  - For each cryovial to be thawed, coat an appropriate number of wells of a six-well plate with hESC-Qualified Matrigel beforehand (depending on the number of cells per cryovial)
  - For each well to be seeded, prepare 2 ml mTeSR Plus, preheated to 37°C, with 10 μM ROCK inhibitor in a 15 ml Falcon tube
  - Thaw out a cryovial of frozen hiPSCs at 37°C until partially defrosted
  - Add 0.5 ml preheated mTeSR Plus into the cryovial and gently resuspend to thaw cells
  - Gently transfer the cells to a 15 ml Falcon tube by pipette and make up to 3 ml with media for each 1 ml cryovial
  - Centrifuge at 200 g for 3 min at RT
  - Discard the supernatant and gently resuspend the cell pellet in prepared mTeSR Plus with ROCK inhibitor
  - Take the 6-well culture plate previously coated with Matrigel from the incubator and discard the coating solution from the well, taking care not to damage the coating on the bottom of the well with the pipette tip
  - Plate the cells into the coated wells of 6-well cell culture plate, using 2 ml of cell suspension in mTeSR Plus with 10 μM ROCK inhibitor per well
  - Maintain the cells at 37°C in a tissue culture incubator supplied with 5% of CO_2_
  - Change media after 24 h to fresh mTeSR Plus without ROCK inhibitor
  - Change the media from then onwards every day or every other day with fresh mTeSR Plus without ROCK inhibitor.

*Note*: hiPSCs are highly sensitive. Thawing the cells quickly and being gentle when resuspending is important for maintaining hiPSC viability and pluripotency.

### Passaging hiPSCs

#### Timing: ∼30 min

3. Passage every three to four days when cells are 70%–80% confluent
  - For each well to be seeded, coat a 6-well plate well with hESC-Qualified Matrigel and prepare 2* *ml of mTeSR Plus media with 10* *μM of ROCK inhibitor
  - Use a brightfield microscope to assess the hiPSC culture for any differentiated cells (if present, see *Troubleshooting: Problem 1*)
  - If some differentiated cells are present, mark the area on the bottom of the plate with a marker, or use a stereomicroscope under sterile conditions, and scratch the cells off using a sterile pipette tip
  - Remove the media in each well using a pipette and discard
  - Wash each well with Dulbecco’s phosphate buffered saline (DPBS) once very gently, then discard DPBS solution
  - Add 1* *ml StemPro Accutase Cell Dissociation Reagent (Accutase) into each well for detachment and disassociation of cells
  - Incubate at 37°C for 3* *min inside the cell culture incubator
  - Pipette 2* *ml of mTeSR Plus media into each well immediately after incubation (the final volume should be 3 times the initial volume of Accutase)
  - If necessary, detach the remaining cells fully from the surface using a cell scraper
  - Transfer cells into a Falcon tube and centrifuge at 200* *g for 3* *min at RT to pellet cells
  - Discard the supernatant from the Falcon tube with centrifuged cells
  - Resuspend the cell pellet in 1* *ml of mTeSR Plus media with ROCK inhibitor
  - After discarding the Matrigel coating solution, pipette 2* *ml of mTeSR Plus with ROCK inhibitor into each Matrigel-coated well
  - Normally you can seed hiPSCs into the Matrigel-coated plate at a 1:10 split ratio, but adjust depending on the original and the desired confluency between 1:6 and 1:12
  - Distribute the cells by gently swirling the plates
  - Incubate hiPSCs in a tissue culture incubator at 37°C supplied with 5% of CO_2_
  - Change the media 24* *h after passaging to fresh mTeSR Plus without ROCK inhibitor
  - Change the media from then onwards every day or every other day using fresh mTeSR Plus without ROCK inhibitor.

*Note*: Check the hiPSCs in culture daily to ensure they are growing optimally and to monitor for unwanted spontaneous differentiation. If any differentiation is present, see *Troubleshooting: Problem 1.*

### Day -1: Inducing embryoid body formation

*Note*: Prior to commencing SKO differentiation, hiPSCs should be healthy, stable in culture, and highly pluripotent.

*Note*: hiPSCs should be passaged at least twice after thawing prior to SKO differentiation: at least once with hESC-qualified Matrigel, and once with Growth Factor Reduced Matrigel. Passaging with hESC-qualified Matrigel allows hiPSCs to recover after thawing. Passaging with Growth Factor Reduced Matrigel immediately before commencing SKO culture reduces the risk of any growth factors interfering with the differentiation process.

#### Timing: ∼1 h 30 min

Prior to inducing differentiation to SKOs, hiPSCs are seeded into Ultra-Low Attachment 96-Well Plates and aggregated in E6 media to induce embryoid body formation.

1. Prepare a suitable volume of Day-1 Embryoid Body Formation Media (Table 1) containing Essential 6 (E6) with 0.5× Antibiotic-Antimycotic and 10* *μM ROCK inhibitor (you will need approximately 10* *ml per 96 well plate to be seeded)
2. When hiPSCs in culture reach 70%–80% confluency, detach the cells with StemPro Accutase Cell Dissociation Reagent (Accutase)
  - Use a brightfield microscope to assess the hiPSC culture for any differentiated cells (if present, see *Troubleshooting: Problem 1*)
  - Remove the media in each well using a pipette and discard
  - Wash each well with DPBS once very gently, then discard DPBS solution
  - Add 1ml of Accutase into each well for detachment and disassociation of cells
  - Incubate at 37°C for 3* *min inside the cell culture incubator
  - Pipette 2* *ml of mTeSR Plus media into each well immediately after incubation (the final volume should be three times the initial volume of Accutase)
  - If necessary, detach the remaining cells fully from the surface using a cell scraper
3. Transfer the hiPSCs into a Falcon tube and centrifuge at 200* *g for 3* *min at RT
4. Discard the supernatant from the Falcon tube with the centrifuged hiPSCs, and resuspend the cell pellet in mTeSR Plus media (use around 5* *ml and adjust if too concentrated)
5. Count the number of hiPSCs in the suspension using either a haemocytometer as described below, or an automated cell counter
  - Pipette 50* *μl of the resuspended cells into an Eppendorf tube
  - Add 50* *μl of Trypan blue into the same tube and pipette up and down a few times for even mixing
  - Place 10* *μl of sample into one part of the haemocytometer and count the number of live cells in two 4* *×* *4 diagonally opposite squares under the microscope using 10× objective
  - For cell counting, multiply this by 10^4^ to get the concentration of cells/ml in your cell suspension (this is the c1 value)
  - Since you need 8000 cells per well for embryoid body formation, with a volume of 100* *μl (0.1* *ml) per well, you will need a final concentration of 80 000* *cells/ml (this is the c2 value)
  - If you are seeding one 96-well plate with a volume of 100* *μl (0.1* *ml) per well, you will need a total volume of approximately 10* *ml cell suspension in E6 (this is the v2 value)
  - Use (c2* *×* *v2)/c1* *=* *v1, where v1 is the volume of your original suspension needed
6. Transfer the calculated volume of cell suspension into a separate Falcon tube and centrifuge at 200* *g for 3* *min at RT
7. Resuspend the hiPSCs in 1* *ml of previously prepared Day-1 Embryoid Body Formation Media, then make up to the correct final volume with the same media
8. Transfer the cell suspension into a multi-channel pipette reservoir
9. Using a 200* *μl multichannel pipette, seed 100* *μl of cells into each well of the Ultra-Low Attachment 96-Well Plates, gently mixing the cells midway so they do not settle to the bottom of the reservoir
10. Centrifuge the plates at 250* *g for 5* *min to aid hiPSC aggregation
11. Incubate at 37°C in a tissue culture incubator supplied with 5% of CO_2_ for 24* *h.

**Table 1.** Day -1 embryoid body formation medium.

Reagent	Final concentration	Amount
Essential 6 Medium	–	10 ml
ROCK Inhibitor	10 µM	10 µl
Antibiotic-Antimycotic	0.5×	50 µl
Total	n/a	∼10 ml

*Note:* SKO differentiation is highly sensitive and can be disrupted by even the routine door opening of standard tissue culture incubators. The SKOs seeded in the outer wells of the 96-well culture plate often differentiate poorly as a result, failing to form the transparent cyst structure shown in Fig. 1. This is a major reason why it is preferable to prepare a surplus of organoids in the initial steps.

**Figure 1.**
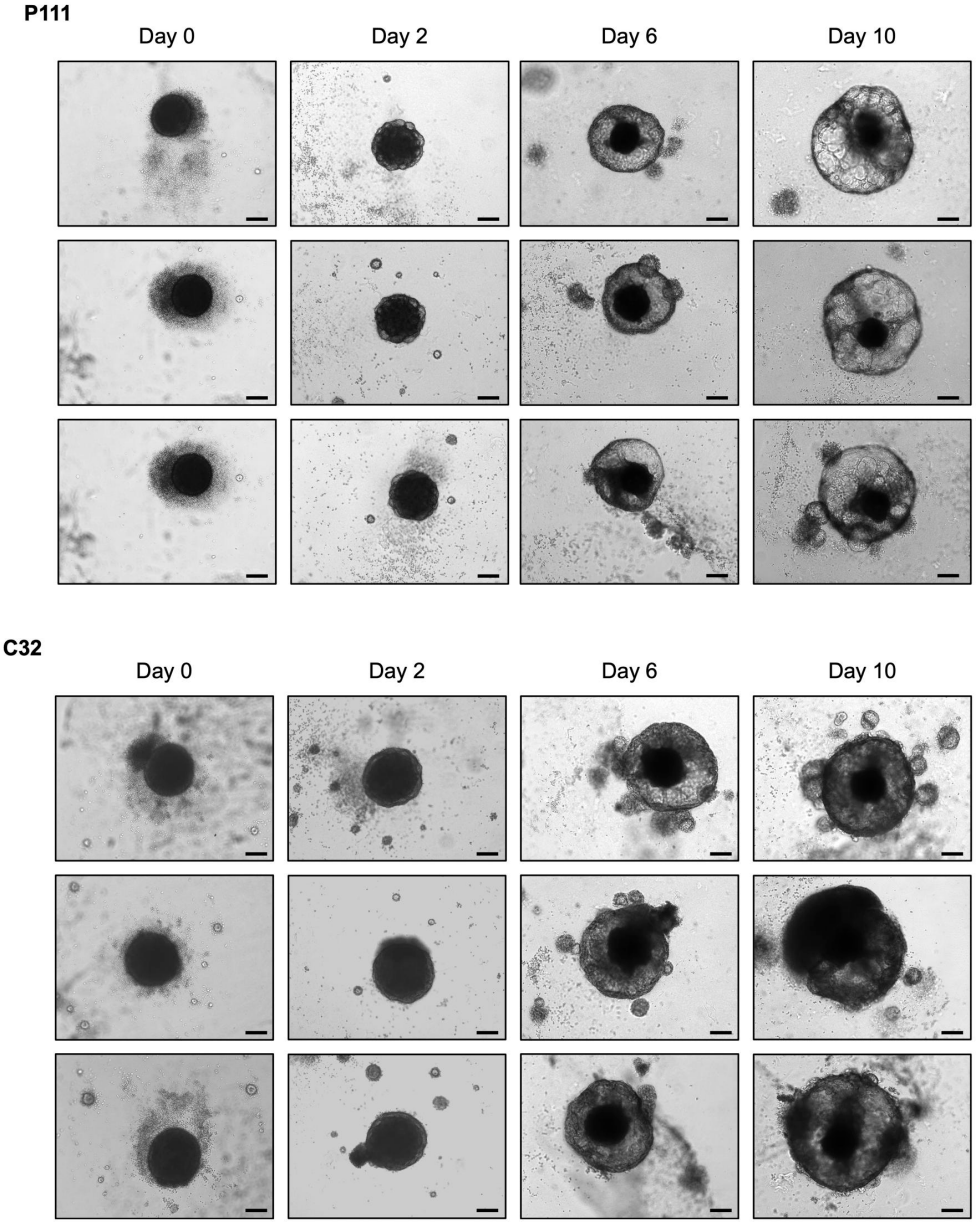
Early differentiation of skin organoids (SKOs) from two human induced pluripotent stem cell (hiPSC) lines. Brightfield images of representative SKOs at Days 0, 2, 6 and 10 of development, derived from the P111 hiPSC line (CD34+ placental cell-derived) and the C32 hiPSC line (skin fibroblast-derived). The embryoid body starts to develop into a transparent epithelial cyst. Scale bar: 200 μm.

### Day 0: Inducing differentiation to non-neural ectoderm

#### Timing: ∼1 h (depending on number of organoids)

To induce the differentiation of non-neural ectoderm from which the skin epidermis can develop, embryoid bodies are treated with media containing a transforming growth factor beta inhibitor (SB), bone morphogenic protein 4 (BMP-4) and low concentration of basic fibroblast growth factor (FGF2).

12. Check embryoid bodies to ensure they have formed properly (for more information, see *Troubleshooting: Problem 2*)
13. Prepare a suitable volume of E6-based Day 0 differentiation medium (Table 2) containing 2% Growth Factor Reduced Matrigel, 10* *µM SB, 4* *ng/ml FGF2, 10* *ng/ml BMP-4 and 0.5× Antibiotic-Antimycotic
  - You will need to prepare 10* *ml per 96-well plate
14. Using a 200* *µl pipette, aspirate 80* *µl medium out of each well of the 96-well culture plates, tilting the plate towards you and aspirating from the sides of the well so as not to disturb the embryoid bodies
15. Use a multichannel pipette to carefully transfer 100* *µl of E6-based Day 0 differentiation medium into each well
16. Incubate at 37°C in a tissue culture incubator supplied with 5% of CO_2_ for 3* *days

**Table 2.** Day 0 differentiation medium.

Reagent	Final concentration	Amount
Essential 6 Medium	98%	9.6 ml
Growth Factor Reduced Matrigel (diluted 1:1 in DMEM)	2%	400 µl
SB431542 (SB)	10 µM	10 µl
Recombinant Human FGF-basic (FGF2)	4 ng/ml	0.4 µl
Recombinant Human BMP-4 (BMP4)	10 ng/ml	2 µl
Antibiotic-Antimycotic	0.5×	50 µl
Total	n/a	∼10 ml

*Note:* The use of an automated liquid handling system is preferable when performing these steps, to increase throughput and reproducibility. However, this step can also be performed manually, as described here. When removing the 80 µl of media from the 96-well culture plates manually, it can be helpful to use a sterile stereomicroscope or have a sterile light source beneath the plate, as it allows the embryoid bodies to be visualized when aspirating the media. Do not use a multichannel pipette to remove media from the 96-well cell culture plates, as these are difficult to control, and you risk damaging or aspirating the embryoid body when inserting the tips for media aspiration.

### Day 3: Inducing differentiation into cranial neural crest cells

#### Timing: ∼30 min

To induce the formation of cranial neural crest cells from which the dermis can differentiate, a BMP inhibitor (LDN) and a higher concentration of FGF2 are added to the media.

17. Prepare E6-based Day 3 differentiation media (Table 3) with 1** **µM LDN, 250** **ng/mL FGF2 and 0.5× Antibiotic-Antimycotic
  - You need a volume of 25** **µl per well, so prepare 2.5** **ml per 96 well plate
18. Use a multichannel pipette to add 25** **µl of E6-based Day 3 differentiation media to each well of the 96-well plate
  - The final volume of media will be 125** **µl per well (accounting for 20** **µl evaporation), with a final concentration of 200** **nM for LDN, and 50** **ng/ml for FGF2
19. Return the plate to the tissue culture incubator and incubate at 37°C with 5% of CO_2_ for 3** **days

**Table 3.** Day 3 differentiation medium

Reagent	Final concentration	Amount
Essential 6 Medium	–	10 ml
LDN193189 (LDN)	1 µM	1 µl
Recombinant Human FGF-basic (FGF2)	250 ng/ml	25 µl
Antibiotic-Antimycotic	0.5X	50 µl
Total	n/a	∼10 ml

*Note:* To avoid damaging the SKOs with the pipette tips when adding differentiation media, you can place the tips against the walls of the wells then eject the media. When using this technique, ensure the media properly enters the culture and does not remain as a separate drop on the wall of the well.

### Days 6–10: Adding fresh E6 media and performing media change

#### Timing: ∼30 min for Day 6, ∼1 h for Days 8 and 10

Adding fresh media and performing half media changes with E6 maintains the SKO culture and allow for ongoing differentiation and maturation.

20. On Day 6, add 75** **µl of fresh E6 medium with 0.5× Antibiotic-Antimycotic to each well using a multi-channel pipette, to give a final volume of 200** **µl
21. On Days 8 and 10, aspirate 100** **µl of media from each well and replace with 100** **µl of fresh E6 with 0.5× Antibiotic-Antimycotic.

### Day 12: Transferring to 24-well cell culture plate

#### Timing: ∼2 h

SKOs are transferred to a 24-well culture plate with 500 µl of maturation media, to promote growth and accommodate increasing size.

22. Check SKOs under a brightfield microscope and mark any that do not look like they have formed proper cysts, so you do not transfer these (for more information, see *Troubleshooting: Problem 3*)
23. Prepare a suitable volume of organoid maturation media (OMM) containing 1% Growth Factor Reduced Matrigel
  - You will need 500** **µl OMM with 1% Growth Factor Reduced Matrigel per well of a 24-well plate
  - For the composition of OMM without Matrigel, see Table 4
  - Filter the OMM using a Rapid-Flow Filter Unit (0.2** **µm pore size)
24. Prepare a suitable number of Ultra-Low Attachment 24-well plates with 500** **µl OMM 1% Growth Factor Reduced Matrigel per well
25. Cut 200** **µl pipette tips with a sterile scissor to widen the orifice and accommodate the SKO
26. Transfer SKOs to OMM by aspirating each organoid in 30–50** **µl media and pipetting it into the Ultra-Low Attachment 24-well plate, with one SKO per well
27. Return the plates to the tissue culture incubator and incubate at 37°C with 5% CO_2_

**Table 4.** Organoid maturation medium.

Reagent	Final concentration	Amount
Advanced DMEM/F12	49%	24.5 ml
Neurobasal Medium	49%	24.5 ml
GlutaMAX™ Supplement	1×	500 µl
B-27 Supplement, Minus Vitamin A	0.5×	500 µl
N-2 Supplement	0.5×	250 µl
2-Mercaptoethanol	0.1 mM	91 µl
Antibiotic-Antimycotic	0.5×	250 µl
Total	n/a	∼50 ml

*Note*: Transfer only SKOs that have formed proper cysts, as these are likely to differentiate well to form skin structures. Use wide orifice pipette tips for the transfer, so as not to damage the SKOs. Discard any skin organoids which have failed to form cysts and appear as a solid, dense mass.

The cyst formation is a key indicator of correct SKO differentiation. We observed some batch-to-batch variability in cyst formation, including slight differences in their sizes. This variability could be mitigated by using hiPSCs from same source and at identical passage numbers, consistent media sourcing and formulations, regular assessment of hiPSCs for their gene expression and genomic stability, and utilization of automated liquid handling systems.

*Optional:* SKO cultures are best incubated on a shaker to evenly distribute nutrients in the media, however, static culture will also generate hair-bearing SKOs.

### Day 15: Half medium change with OMM with 1% Matrigel

#### Timing: ∼1 h (depending on the number of organoids)

A half media change is performed to add fresh media and provide nutrients for the developing SKOs.

28. Prepare a suitable volume of OMM with 1% Growth Factor Reduced Matrigel
29. Aspirate 250** **µl of spent media from the well and replace it with 250** **µl fresh OMM with 1% Growth Factor Reduced Matrigel

### Day 18 onwards: Half medium changes with OMM

#### Timing: ∼1 h (depending on the number of organoids)

Half media change is performed to add fresh media and provide nutrients for the developing SKOs.

**Figure 2.**
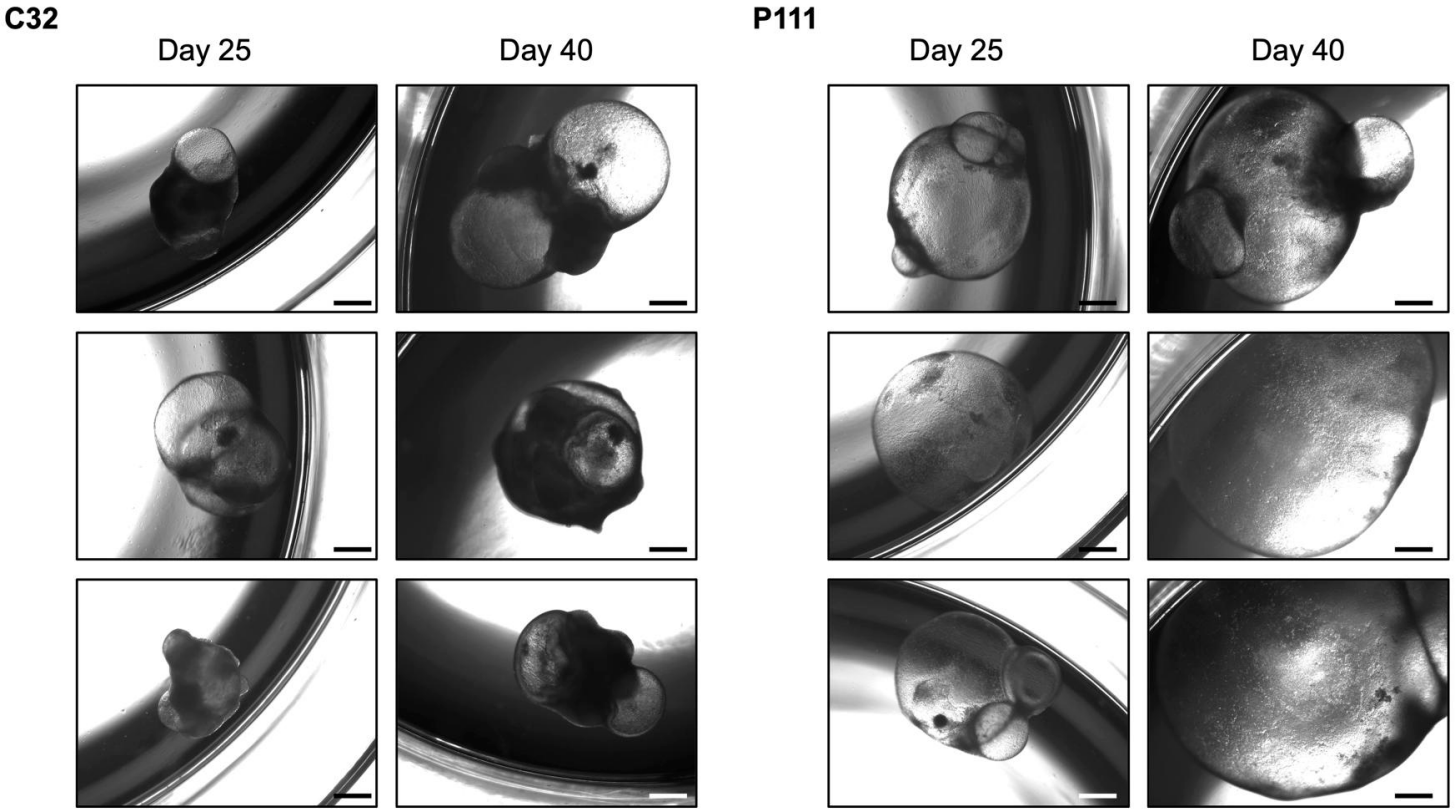
Growth and maturation of skin organoids (SKOs) from two human induced pluripotent stem cell (hiPSC) lines. Brightfield images of representative SKOs at Days 25 and 40 of development, derived from the C32 and P111 hiPSC lines. SKO morphology varies between organoids from different hiPSC lines and within hiPSC lines, however cyst formation remains a key indicator of correct differentiation. Scale bar: 500 μm.

**Figure 3.**
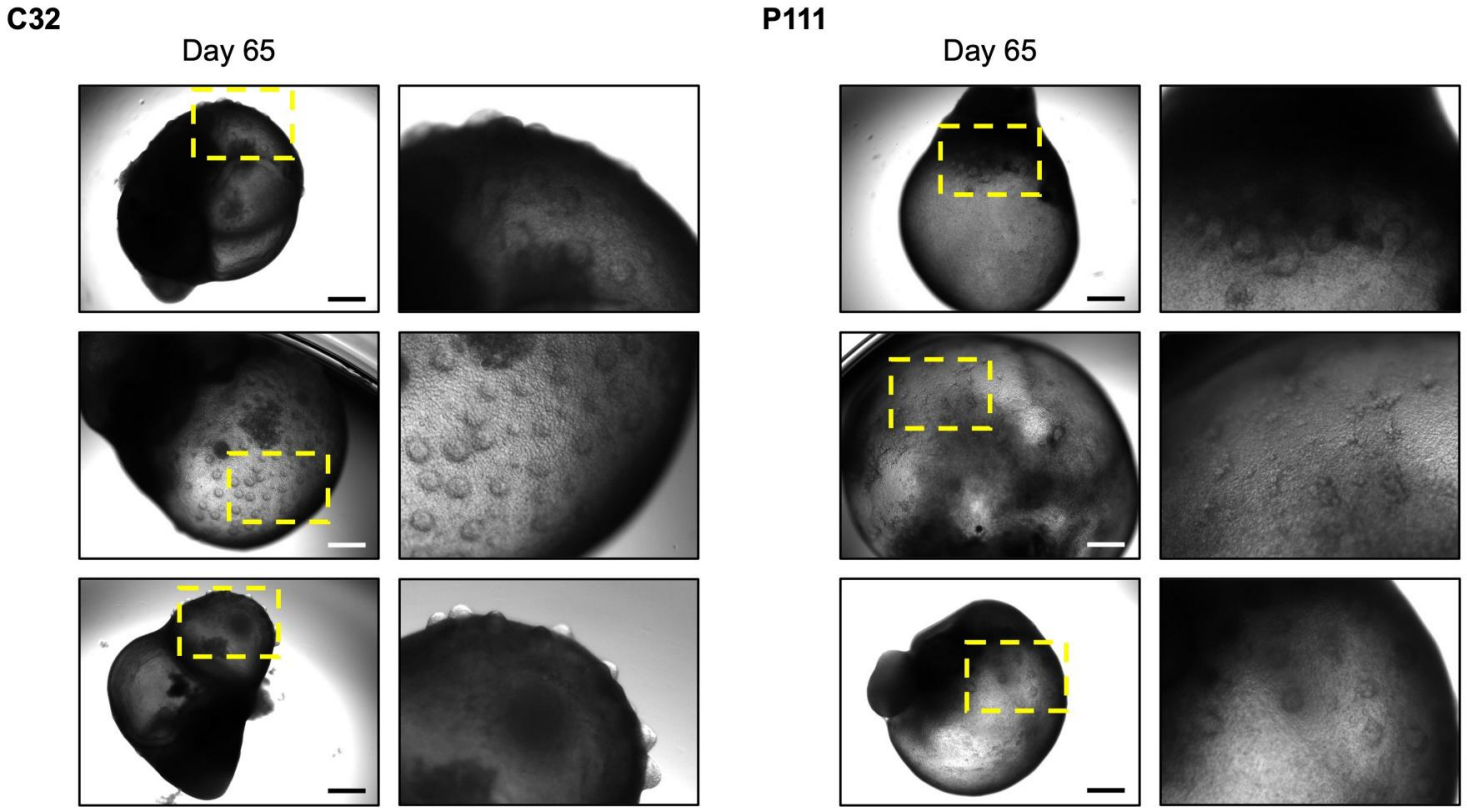
Development of hair follicles in skin organoids (SKOs) from two human induced pluripotent stem cell (hiPSC) lines. Brightfield images of representative SKOs at Day 65 of development, derived from the C32 and P111 hiPSC lines. Early hair follicles are identifiable as circular clusters of cells that start to protrude from organoid surface. Dashed box indicates magnified region. Scale bar: 500 μm.

**Figure 4.**
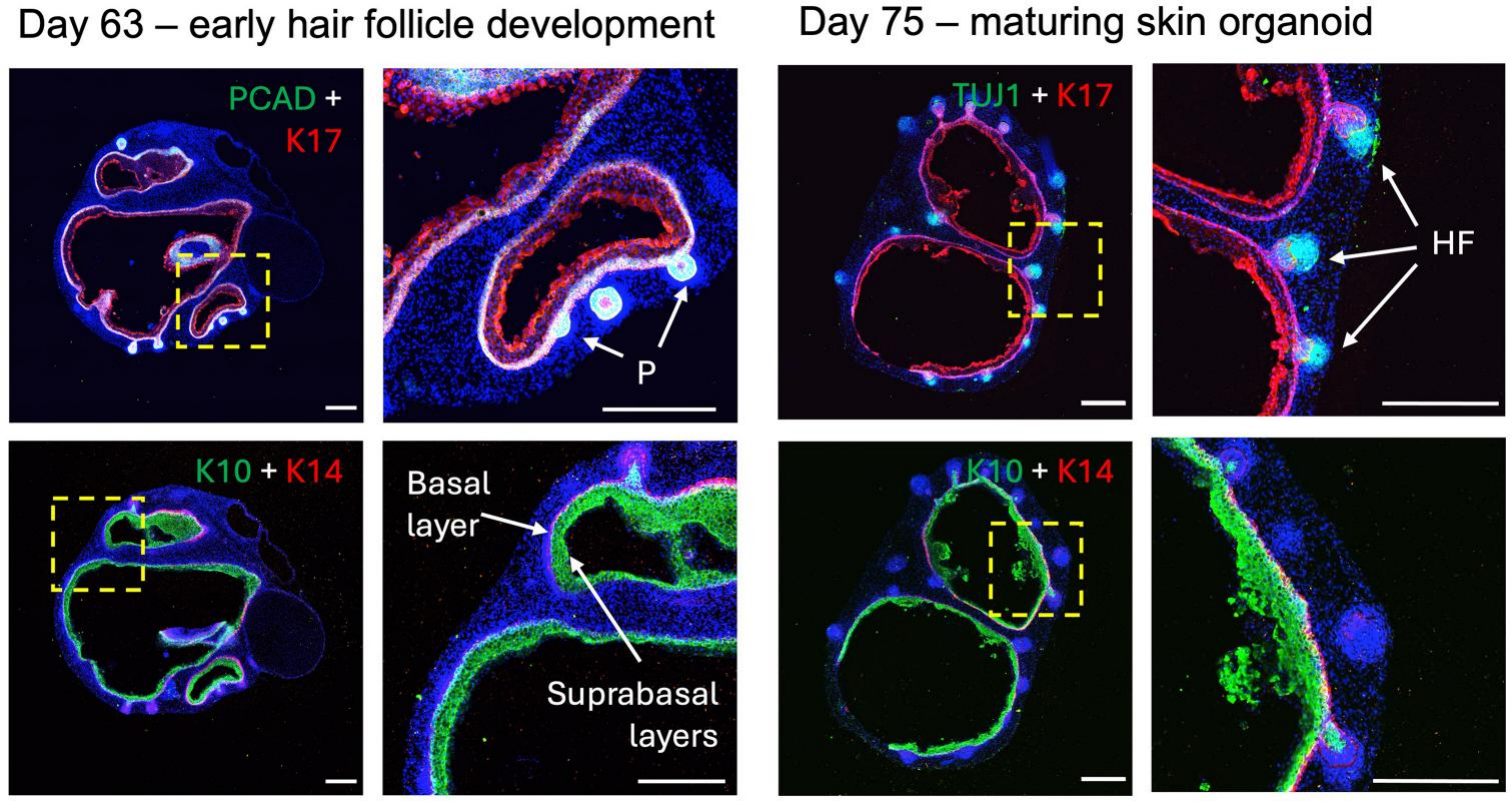
Immunofluorescence analysis of skin organoids (SKOs) derived from the P111 human induced pluripotent stem cell (hiPSC) line at different stages of maturation. Images of representative sectioned SKOs at Days 63 and 75 of development, stained with various fluorescent markers. K14 staining indicates the epidermis basal layer, and PCAD marks both the basal layer and hair placodes. K10 indicates epidermal suprabasal layers. K17 indicates proliferative keratinocytes and the HF outer root sheath. TUJ1 marks neurites. Blue indicates cell nuclei stained with DAPI. Dashed yellow boxes indicate magnified regions. HF, hair follicle; P, hair placode. Scale bar: 300 μm.

*Note*: Judge whether to increase the volume of media per well for the SKOs based on the organoid size and the colour of the media. If the media is turning yellow very rapidly, it indicates that the media volume is insufficient and needs increasing.

30. Half medium change is performed every three days or every other day using fresh OMM without Matrigel
31. The medium volume in each well is increased up to 1.2** **ml from Day 80 onward as SKOs mature and grow larger (see Figs 2 and 3 for reference regarding organoid growth and development, and Fig. 4 as reference for SKO immunofluorescence analysis)

## Expected outcomes

### Limitations

We have used this protocol to generate hair-bearing SKOs with two hiPSC lines from different somatic sources, specifically skin fibroblasts (C32 hiPSCs) and CD34+ placental cells (P111 hiPSCs). This demonstrates that this protocol is successful across multiple hiPSC lines with different somatic sources [7]. However, there remains the possibility that this protocol is not successful in all hiPSC lines. In a situation where SKO differentiation is unsuccessful, as indicated by lack of cyst formation or absence of skin marker expression and appendage formation, performing sequential optimization of the concentrations of the differentiation factors, specifically BMP-4 at Day 0, and LDN and FGF2 at Day 3, is recommended as described previously [6].

A key limitation of current SKOs differentiation protocols is the lengthy culture process needed to obtain a mature, appendage-bearing SKO. Current differentiation protocols require approximately 60 days in culture until the organoids robustly develop hair follicles, and sweat gland structures are evident at Day 90 of culture. This is because current differentiation protocols [5, 7] mimic the process of embryogenesis, and therefore the necessary time to generate skin structures reflects the time taken for them to develop within an embryo. Although this method is advantageous in that the skin produced closely recapitulates bona fide foetal skin, the lengthy culture process creates a risk for culture contamination. As such, we recommend taking every care to use sterile consumables, to filter the media, and to clean instruments with appropriate disinfecting methods when performing media changes, to minimize the chance of culture contamination. This is especially critical if the aim is to do SKO transplantation studies.

Another limitation of SKO differentiation protocols is that the SKOs produced are lacking a few of the key features of bona fide human skin, specifically immune cells, hair follicle cycling, and vasculature. This may affect the SKOs fidelity as an *in vitro* model for drug testing, depending on whether the drug interacts with and affects any of these features within the skin. The lack of vasculature may also be a limiting factor in SKO maturity. Indeed, cortical brain organoids with vasculature were found to have enhanced functional maturation as compared to their non-vascularized counterparts [8]. Furthermore, incorporating vasculature into the SKO could improve engraftment upon transplantation. There are several approaches that have been used to introduce a vascular network into organoids and could be applied to generate vascularized SKOs, however, until this is achieved, the lack of vasculature remains a limiting factor in the SKO’s ability to recapitulate bona fide skin. Additionally, some off-target differentiation leading to the development of chondrocytes within the SKOs is observed with current differentiation protocols, highlighting the need for further optimization of the SKO differentiation process [6, 7].

Current protocols for generating SKOs are also limited by the cystic architecture of the skin produced. Unlike bona fide skin, which develops in a planar fashion, the SKO develops as an inside-out cyst with the epidermis forming on the inner layer and the dermis on the outer layer. This means that hair follicle development occurs with the dermal papilla forming on the outer surface of the cyst and the hair growing inward. Furthermore, there is a great deal of variability between individual SKOs, in terms of size, morphology, and the number of appendages. Using a culture method which pools organoids together in the media, such as a spinning bioreactor, may generate SKOs with less variability than using the separate welled culture plate technique described here.

## Troubleshooting

### Problem 1: Spontaneous and off-target differentiation of hiPSCs in culture

**Figure 5.**
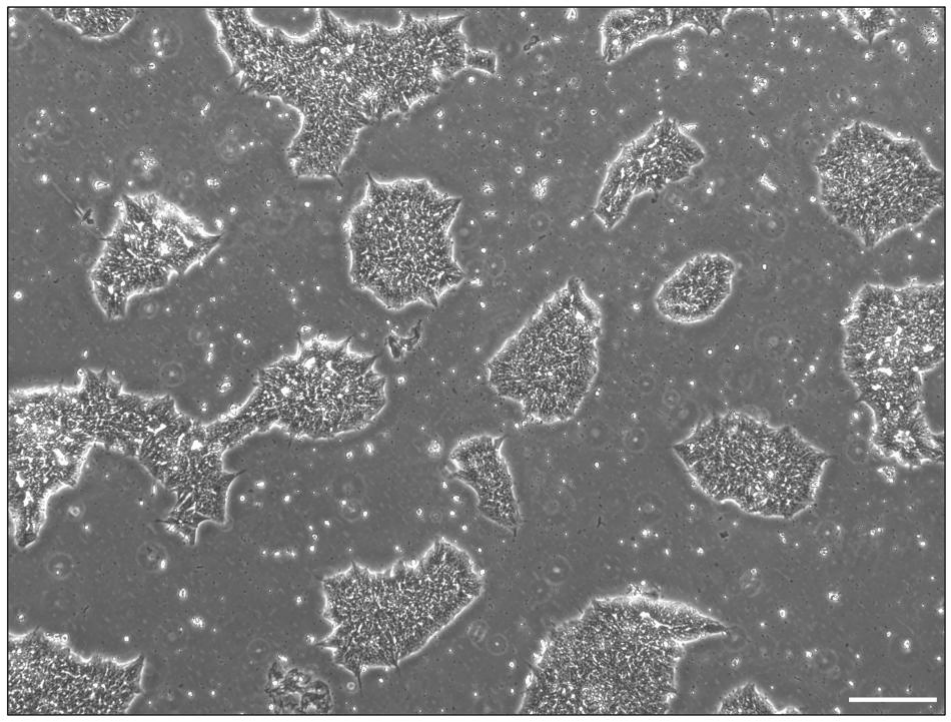
Human induced pluripotent stem cell (hiPSC) colonies in culture with mTeSR Plus. Brightfield image of P111 hiPSCs in culture at approximately 50% confluency. hiPSCs exhibit decent quality as there are no differentiating cells around the edges of the colonies. Scale bar: 200 μm.

hiPSCs are sensitive cells which, if not maintained properly, can start to undergo spontaneous differentiation in culture. Differentiated cells often develop around the edges of hiPSC colonies and can be identified by using a brightfield microscope to observe cell morphology. An image of healthy hiPSC colonies is shown in Fig. 5.

#### Potential solution

Prior to passaging, if there is a small patch of differentiated cells within the hiPSC culture, a brightfield microscope and a permanent pen can be used to mark the differentiated region on the bottom of the 6-well plate. Then, the differentiated cells can be gently scraped using a sterile pipette tip within a biosafety cabinet. If a stereomicroscope is available under sterile conditions, this can alternatively be used to identify and remove any differentiated patches. The differentiated cells, now floating in the media, will be removed during the DPBS wash step.

However, if there are many differentiated cells within the culture, it can be difficult to salvage and it is better to discard the cells and thaw out a new cryovial of hiPSCs. To help maintain hiPSC pluripotency, be gentle when resuspending the hiPSCs, passage them before they become overconfluent, and do not keep the culture out of the incubator for more than 15 min at a time.

### Problem 2: Poor embryoid body formation

**Figure 6.**
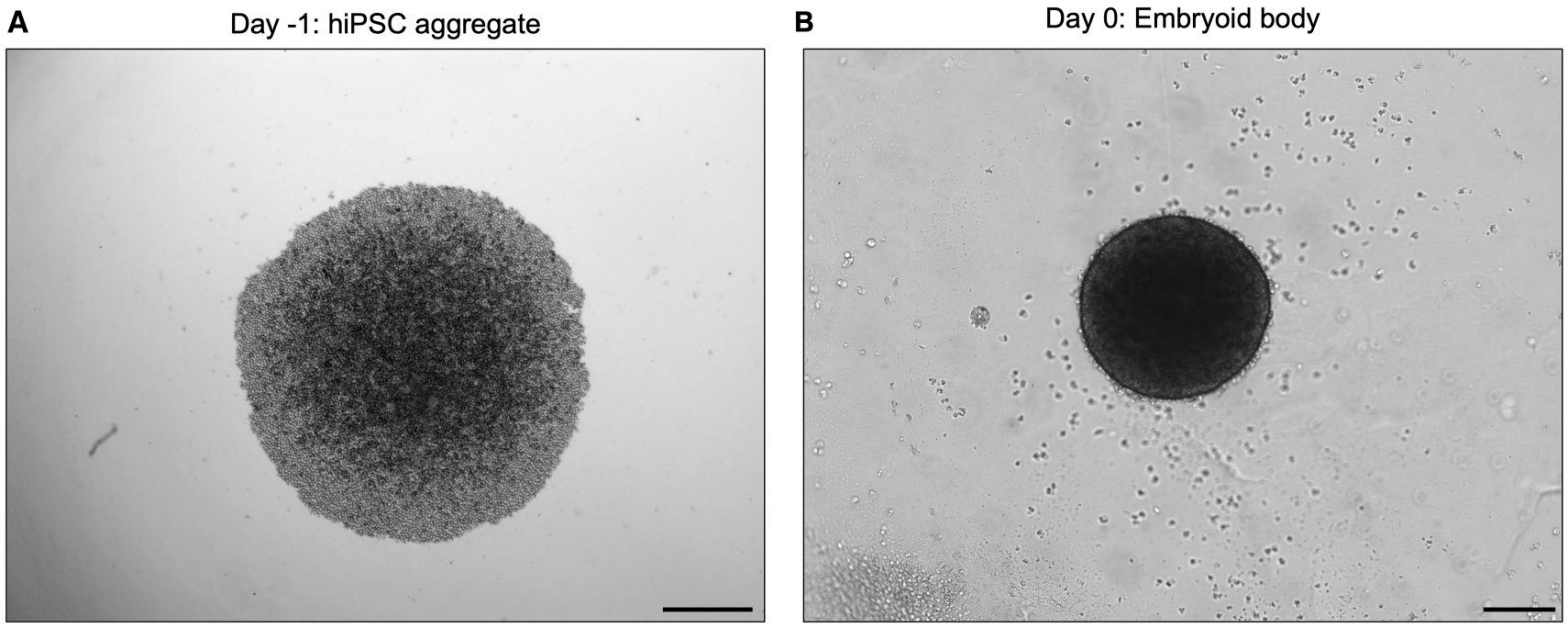
Correct morphology of the human induced pluripotent stem cell (hiPSC) aggregate (A) and the embryoid body (B). Brightfield images of a representative hiPSC aggregate at Day -1 after the plate centrifugation (A) and the embryoid body at Day 0 (B), both derived from the P111 hiPSC line. Scale bars: 500 μm (A) and 200 μm (B).

Good quality embryoid bodies have a dense centre and a smooth, defined edge as shown at Day 0 in Figs. 1 and 6B. If at Day 0, the embryoid body has rough edges or cyst-like regions, it is likely that embryoid body formation has not occurred properly.

#### Potential solution

To prevent this problem from arising, only use reagents that adhere to established quality control standards and ensure they have been stored appropriately. Ensure that the hiPSCs used are healthy and pluripotent (as indicated by their morphology and expression of pluripotency markers), and that they are not over or under-confluent prior to starting the Day -1 protocol. Throughout the Day -1 protocol, do not triturate the hiPSCs too harshly with the pipette, as this can be damaging. After the final centrifugation of the 96-well plates with the hiPSCs on Day -1, use a brightfield microscope to check that the hiPSCs have aggregated appropriately. The hiPSCs within the wells should appear as a circular aggregate as shown in Fig. 6A. If the cells have not aggregated together, it is likely that centrifugation of the culture plates did not occur properly.

### Problem 3: Lack of cyst development in early SKO culture

After Day 0 differentiation, the SKO develops into an epithelial cyst, visible as a transparent cyst under brightfield microscope as shown in Fig. 1. If, however, the SKO remains as a dense, solid mass, it indicates that the differentiation has not occurred properly and that the skin structures have not formed. This is especially common for the organoids seeded into the boundaries of the 96-well plate, likely due to the increased evaporation affecting the concentrations of differentiation factors in the media for those wells.

#### Potential solution

To minimize issues with differentiation, it is important to follow the protocol steps carefully, ensure that the reagents used are in good condition, and that the initial embryoid bodies are of good quality. When commencing a new batch of SKOs, it is also best to prepare a surplus in the 96-well plates, as there are often a few organoids which do not develop proper cysts by Day 12, such as the organoids on the boundary wells. Alternatively, we have found that using an incubator with segmented inner doors greatly improves the differentiation of SKOs at the plate boundary region, as opposed to using a standard tissue culture incubator. This likely due to the improved regulation of temperature, gas concentration, and humidity.

### Problem 4: Hair follicles not visible on mature SKO

**Figure 7.**
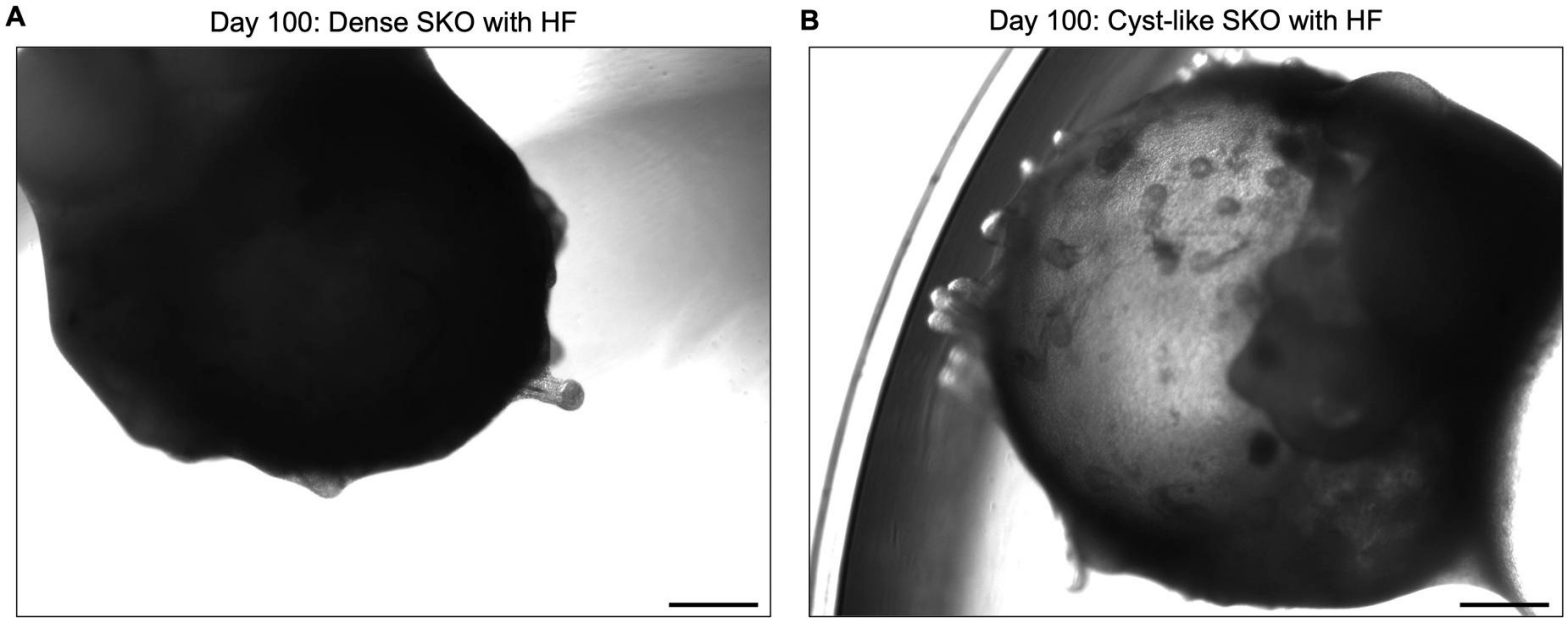
Visualizing hair follicles on denser skin organoids (SKOs) is more difficult than in more transparent cyst-like SKOs. Brightfield images of representative SKOs at Day 100 with denser structure (A) and a more transparent cyst (B), both derived from the P111 human induced pluripotent stem cell (hiPSC) line. In A, only a few hair follicles protruding from the SKO surface are visible, as the dense structure obscures any other follicles on the organoid surface, whereas in B, hair follicles over the entire organoid surface can be visualized. Scale bars: 500 μm.

Often in later stages of culture, the SKOs appear denser due to the increasing thickness of the skin layers, as shown in Fig. 7A. This can make hair follicles difficult to visualize on the SKO surface when using a brightfield microscope, as compared to SKOs with a more transparent cyst (Fig. 7B).

#### Potential solution

By sectioning SKOs, the hair follicles become identifiable, especially when performing immunostaining for hair follicle markers (Fig. 4). Alternatively, dark-field imaging can make the hair follicles easier to visualize in live organoids, especially if the follicles have developed pigmentation. A small portion of SKOs simply do not develop hair follicles as they mature, due to the intrinsic variability between individual organoids. Therefore, we recommend marking the SKOs with hair follicles when the follicles first develop, so at later timepoints when the follicles are harder to visualize, there is an indicator of which organoids are hair-bearing and which are not.

## Data Availability

The data underlying this article are available in the article.
